# A mini-review: phosphodiesterases in charge to balance intracellular cAMP during T-cell activation

**DOI:** 10.3389/fimmu.2024.1365484

**Published:** 2024-03-08

**Authors:** Marie Bielenberg, Roberta Kurelic, Stefan Frantz, Viacheslav O. Nikolaev

**Affiliations:** ^1^ Department of Internal Medicine I, University Hospital Würzburg, Würzburg, Germany; ^2^ Institute for Experimental Cardiovascular Research, University Medical Center Hamburg-Eppendorf, Hamburg, Germany; ^3^ German Center for Cardiovascular Research (DZHK), partner site Hamburg/Kiel/Lübeck, Hamburg, Germany; ^4^ Comprehensive Heart Failure Center, University Hospital Würzburg, Würzburg, Germany

**Keywords:** cAMP, phosphodiesterases, T-cell activation, T-cell subsets, cGMP-to-cAMP crosstalk, PDE2A

## Abstract

T-cell activation is a pivotal process of the adaptive immune response with 3′,5′-cyclic adenosine monophosphate (cAMP) as a key regulator of T-cell activation and function. It governs crucial control over T-cell differentiation and production of pro-inflammatory cytokines, such as IFN-γ. Intriguingly, levels of intracellular cAMP differ between regulatory (Treg) and conventional T-cells (Tcon). During cell-cell contact, cAMP is transferred via gap junctions between these T-cell subsets to mediate the immunosuppressive function of Treg. Moreover, the activation of T-cells via CD3 and CD28 co-stimulation leads to a transient upregulation of cAMP. Elevated intracellular cAMP levels are balanced precisely by phosphodiesterases (PDEs), a family of enzymes that hydrolyze cyclic nucleotides. Various PDEs play distinct roles in regulating cAMP and cyclic guanosine monophosphate (cGMP) in T-cells. Research on PDEs has gained growing interest due to their therapeutic potential to manipulate T-cell responses. So far, PDE4 is the best-described PDE in T-cells and the first PDE that is currently targeted in clinical practice to treat autoimmune diseases. But also, other PDE families harbor additional therapeutic potential. PDE2A is a dual-substrate phosphodiesterase which is selectively upregulated in Tcon upon activation. In this Mini-Review, we will highlight the impact of cAMP regulation on T-cell activation and function and summarize recent findings on different PDEs regulating intracellular cAMP levels in T-cells.

## 1 Introduction

Since its initial discovery in 1957, multifaceted roles in different cell types have been discovered for the second messenger 3′,5′-cyclic adenosine monophosphate (cAMP) (1). Importantly, cAMP as pivotal regulator of the adaptive immune system exerts control over T-cell activation, differentiation, and the production of pro-inflammatory cytokines like IFN-γ (2, 3). cAMP is generated from adenosine triphosphate (ATP) by adenylyl cyclases (ACs). Initiation of cAMP synthesis is facilitated by the binding of extracellular ligands, including cytokines, catecholamines, and adenosine, to various stimulatory G-protein coupled receptors (GPCRs). Ligand binding induces a conformational change of the GPCR and subsequent dissociation of G_α_ and G_βγ_-subunits. G_α_ binds to ACs, leading to generation of cAMP (4). Among 10 mammalian AC isoforms, AC1-9 are membrane-bound and only AC10 is known to be soluble (5). In T-cells, AC7 is the predominant isoform, although AC3, AC6 and AC9 are also expressed (6, 7). The knock-out of AC7 in the hematopoietic system of mice exhibits a reduced total number of leukocytes and an impaired immune response against T-cell dependent antigens (7). Elevated intracellular cAMP levels activate several effector molecules. Notably, the binding of cAMP to the protein kinase A (PKA) is the best-characterized interaction downstream of the cAMP signaling pathway. The induced dissociation of the two regulatory subunits of PKA enables the phosphorylation of threonine and serine residues in various proteins e.g., cAMP-response element binding protein (CREB), cAMP-response element modulator/inducible cAMP early repressor (CREM/ICER) or the nuclear factor-κB (NF-κB) (8–10). High levels of cAMP are balanced by phosphodiesterases (PDEs), a superfamily of enzymes with the ability to degrade cyclic nucleotides, cAMP or cyclic guanosine monophosphate (cGMP) to 5’-AMP and 5’-GMP, respectively (11). Over 100 different isoforms are described in mammals (12). The distinct expression of PDEs, ACs and the A-kinase anchoring proteins enable to formation of local cAMP pools, allowing compartmentalization of cAMP signaling within micro- or nanodomains rather than eliciting a global response within the cell (13).

## 2 cAMP in different T-cell subsets

Naïve T-cells can differentiate into various T-cell subsets with a specific expression pattern of cytokine receptors after their contact with antigens presented by antigen-presenting cells (APCs). Ninety-five percent are conventional T-cells (Tcon) whereas the other five percent of the T-cell population are regulatory T-cells (Treg) expressing the transcription factor Forkhead box protein-3 (*Foxp3*), known to be indispensable for proper Treg development and function (14, 15). Tregs can either be derived from the thymus, or differentiated afterwards. Mice lacking Tregs develop a severe autoimmune disorder (16). Research in the last few years has been focused on how Tregs are able to fulfill their immunosuppressive function. Among other mechanisms, the cAMP signaling pathway plays a crucial role. Tregs harbor much higher cAMP levels compared to Tcon, which can be further increased after T-cell activation (17). The Treg- specific transcription factor *Foxp3* governs the ability to downregulate PDE3B and subsequently block cAMP degradation by PDE3B in Treg (18). Concurrently, the single microRNA miR-142-3p, which is selectively expressed in Treg, elevates the cAMP levels by enhancing the expression of AC9 (6). The knock-out of miR-142-3p in murine T-cells leads to elevated gene expression related to the IFN-γ signaling pathway and high production of IFN-γ (19). Moreover, treatment of different T-cell subsets with IL-2 uncovers the upregulation of AC7 in Tregs and the downregulation in Tcon (20). Secondly, Treg express CD39 and CD73, ectoenzymes on the cell surface with the ability to convert ATP to adenosine (ADO). Subsequently, ADO activates A_2A_R on Tcon and APCs, which increases cAMP production (21). Lastly, there are no extracellular receptors known for cAMP, but cAMP can be transferred from Treg to Tcon via gap junctions. Co-culture of both T-cell subsets leads to increased cAMP levels in Tcon (17). This transfer and increase of cAMP influence the nuclear localization of CREM/ICER in activated T-cells and decrease IL-2 production, a cytokine presented to Tregs to increase its suppressive activity (22).

## 3 cAMP during T-cell activation

APCs present pathogen fragments in a complex with major histocompatibility complex I or II (MHCI/II) to naïve T-cells for activation. These fragments stimulate T-cell receptors (TCR) leading to multiple intracellular signaling cascades. Additionally, a costimulatory signal of CD28 binding to B7.1/B7.2 is required for T-cell activation and Interleukin-2 (IL-2) synthesis (23, 24). Secreted IL-2 binds to IL-2 receptors on T-cells to promote T-cell differentiation. T-cell activation leads to transiently elevated cAMP levels (25). After stimulation of TCR, cAMP is produced in lipid rafts, which is followed by an increased raft-associated PKA activity (26). Among others, the C-terminal Src kinase (Csk) is activated by PKA phosphorylation and inhibits the activity of the lymphocyte-specific protein tyrosine kinase Lck, subsequently leading to downregulation of T-cell receptor signaling (13, 27). However, targeting of regulatory PKA subunit RIα by binding to Ezrin, an A-kinase anchoring protein, is needed for the transport to lipid rafts and for the inhibition of the T-cell activation by the PKA-Csk pathway (28). Additionally, the co-stimulation of CD28 plays a distinct role in balancing TCR-induced cAMP production. In CD3 and CD28 co-stimulated cells, lipid raft-associated PDE4 activity is increased. CD28 mediates the recruitment of a β-arrestin/PDE4D complex to lipid rafts to enhance cAMP degradation (26). β-arrestins are inhibitors of activated GPCRs and terminate their signal transduction. Hence, increased PDE4 activity leads to an inhibitory feedback loop and lower cAMP levels (29). Interestingly, the recruitment of β-arrestin/PDE4 to lipid rafts is also regulated by PI3K (30). Stimulation of CD28 increased PIP3 production via enhanced PI3K activity and β-arrestin can interact with PIP3 via the PH domain-containing protein (PKB). The siRNA mediated knock-down of β-arrestin 1 and 2 in primary T-cells leads to decreased IL-2 and IFN-γ production (30). Recently, it has been shown that high levels of cAMP upregulate the cytotoxic T-lymphocyte-associated protein 4 (CTLA-4) in Tcon and inhibit the binding of CD28 (31).

## 4 Physiological role of Phosphodiesterases in T-cells

Phosphodiesterases (PDEs), a superfamily of 11 enzyme families (PDE1-11), are responsible for balancing intracellular cyclic nucleotide levels. PDEs have different affinities for cAMP or cGMP. PDE 5, 6 and 9 hydrolyze only cGMP, whereas PDE4, 7 and 8 can selectively bind cAMP, and PDE1-3, 10 and 11 degrade both cyclic nucleotides. Importantly, the activity of dual-substrate PDEs can be influenced by the binding of cyclic nucleotides. In T-cells, several PDEs (PDE1-5, PDE7-8, PDE11) have been described.

### 4.1 PDE1

PDE1 with three different subfamilies PDE1A-PDE1C, is the only PDE family, which is known to be activated by calcium and calmodulin via its N-terminal calmodulin binding domain. In human T-cells, no PDE1 expression is detected on mRNA level, but it is inducible by activation via CD3/CD28 co-stimulation, and inhibition of PDE1 suppresses IL-13 production (32).

### 4.2 PDE2A

PDE2A is a unique subfamily with the ability to degrade both cAMP and cGMP. Three different splice variants of PDE2A with different N-terminal domains and different cellular localization are described: PDE2A1 is localized in the cytosol, PDE2A2 in mitochondria and PDE2A3 at the plasma membrane. After the discovery of PDE3 and PDE4 as the main PDEs in T-cells, the regulation of cAMP through PDE2A in T-cells was not in the focus of the research (33). But recently, PDE2A has been found in murine T-cells to be upregulated during activation of Tcon but not Treg (34).

### 4.3 PDE3

As mentioned above, PDE3B is inhibited in Treg by Foxp3 expression (18). A key finding to explain the elevated cAMP levels in this T-cell subset. Interestingly, inhibition of PDE3 in murine as well as in human T-cells led to differentiation of fully functional T-cells. Thus, PDE3 seems to be dispensable, but favoring for T-cell function since these Tregs harbor the potential to prevent allograft rejection (35).

### 4.4 PDE4

PDE4 is the best-characterized PDE. It comprises four different subfamilies PDE4A-PDE4D with multiple individual isoforms and distinct N-terminal domains. PDE4A, PDE4B and PDE4D are predominant subfamilies in T-cells (36). Notably, PDE4B is activated by T-cell receptor (TCR) signaling and controls IL-2 production (37). β-arrestin is able to form a complex with PDE4, which is recruited to lipid rafts after T-cell activation to balance cAMP levels and block PKA activity (26, 27, 38). Driver of the recruitment is CD28 stimulation (26). Three different PDE4 inhibitors, Rofumilast, Apremilast and Crisaborole, are approved to treat chronic obstructive pulmonary disorder (COPD), psoriasis and moderate atopic dermatitis, respectively (39).

### 4.5 PDE7

PDE7 is divided in its two different subfamilies, PDE7A and PDE7B, but only PDE7A is localized in the Golgi-apparatus of T-lymphocytes (40). PDE7A1 and PDE7A3 are upregulated during T-cell activation (41), which has been described also on mRNA level (32). Mice lacking PDE7 have normal T-cell function, indicating that PDE7 is dispensable for T-cell function. Nevertheless, PDE7 inhibitors can suppress T-cell proliferation by elevating cAMP levels (42, 43).

### 4.6 PDE8

The PDE8 can be subdivided into PDE8A and PDE8B subfamilies. For murine T-cells, expression of PDE8A is increased in Tcon. Activation of human T-cells is associated with an upregulation of PDE8A1 (41). In particular, the PDE8A subfamily controls T-cell motility due to the interaction of PDE8 and Raf-1 (44). The research conducted over the years focusing on the role of PDE8 on T-cell function was reviewed more in detail recently (45). Notably, the PDE8 inhibitor PF-04957325 is able to reduce the inflammatory lesion formation in the central nervous system in the experimental autoimmune encephalomyelitis (EAE) mouse model for multiple sclerosis (46). **Table 1** summarizes previous findings about PDE expression and regulation during T cell activation.

**Table 1:** Overview of changes in PDE expression depending on the T-cell subset, activation status and the function of the PDE family in T-cells.

PDE subfamily	Changes during T-cell activation	Treg vs. Tcon	Function	References
PDE1B	↑	–	Activated by calcium	(32)
PDE2A	↑ Tcon only	Higher expression in activated Tcon	Negative cGMP-to-cAMP cross-talk	(33, 34)
PDE3B	Similar Expression in naïve and activated T-cells	FoxP3 inhibits PDE3B expression in Treg	Positive cGMP-to-cAMP cross-talk	(18, 35)
PDE4B/D	↑	Higher expression in Tcon	IL-2 production; β-arrestin/PDE4 complex recruitment to lipid rafts	(26, 27, 33, 36–38)
PDE7A	↑	–	IL-2 production; T-cell proliferation	(32, 40–43)
PDE8A	↑	Higher expression in Tcon	T-cell motility	(41, 44, 45)

↑, denotes upregulation.

## 5 cGMP-to-cAMP cross-talk via PDE2A/PDE3B in T-cells

**Figure 1:**
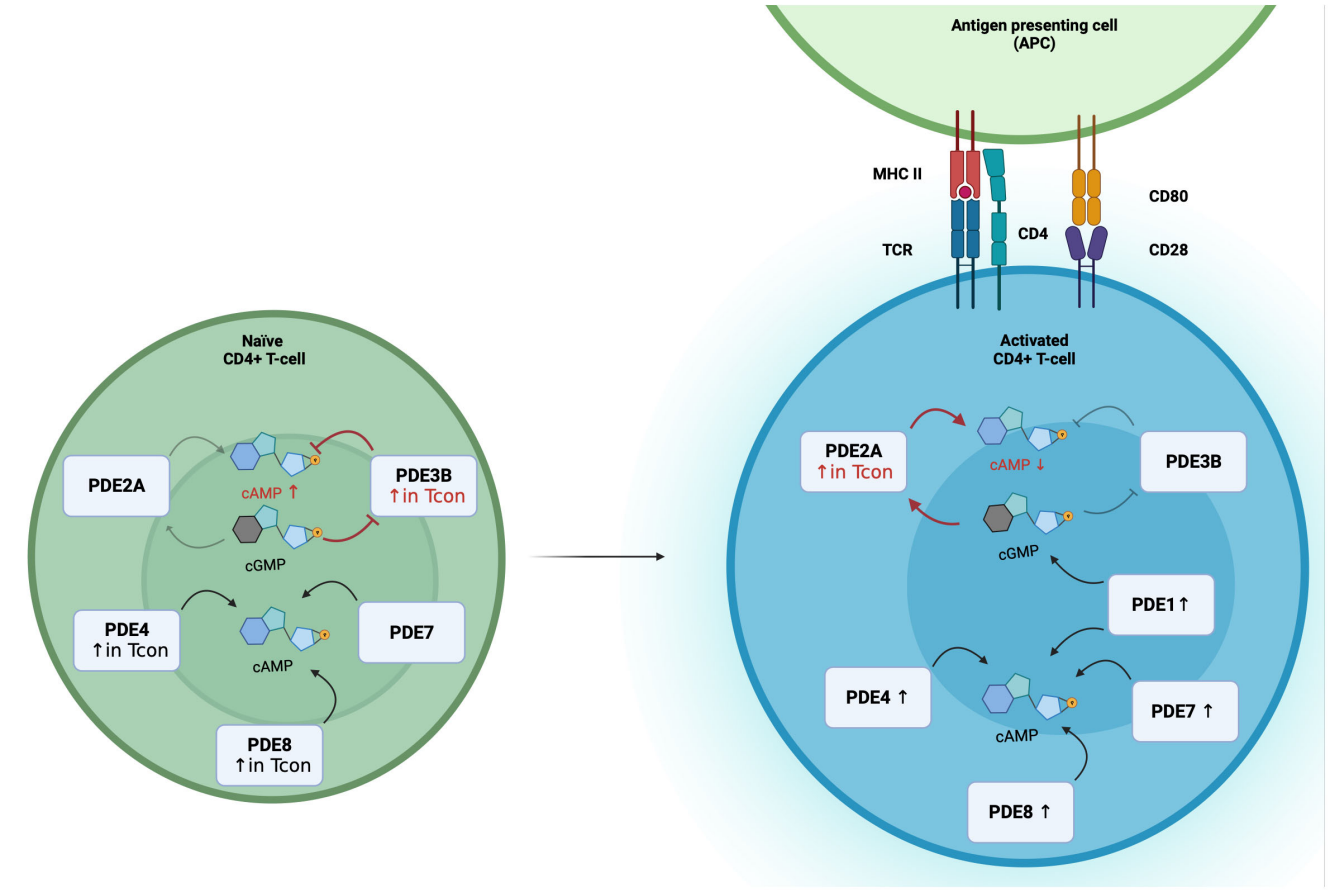
Differential PDE expression and the switch of the cGMP-to-cAMP cross-talk during T-cell activation. In Naïve CD4+ T-cells, PDE2A, PDE3B, PDE4, PDE7 and PDE8 are expressed, with higher expression of PDE3B, PDE4 and PDE8A in Tcon. During T-cell activation PDE1, PDE2A, PDE4, PDE7 and PDE8 expression are upregulated. In Tcon, the cGMP-to-cAMP switches during activation. In naïve Tcon, cGMP binds to PDE3B and acts as a competitive inhibitor of cAMP hydrolysis to enable the positive cGMP-to-cAMP cross-talk. During activation, elevated PDE2A levels result in the negative cGMP-to-cAMP cross-talk. Binding of cGMP to PDE2A leads to a higher cAMP degradation.

cGMP formation is triggered by binding of natriuretic peptides (NPs) to guanylyl cyclases: atrial (ANP) and brain natriuretic peptides (BNP) bind to the guanylyl cyclase-A, C-type natriuretic peptides to guanylyl cyclase-B. Both NP receptors harbor an intracellular guanylyl cyclase domain and are also called particulate guanylyl cyclases (pGC). Alternatively, cGMP formation is catalyzed by nitric oxide sensitive or soluble (sGC) inside the cell. Dual-substrate phosphodiesterases, PDE1-PDE3, PDE10 and PDE11, allow the cross-talk between both cyclic nucleotides. PDE2A has a K_m_ value of 30 µmol/L for cAMP and 10 µmol/L for cGMP hydrolysis, and is also called cGMP-stimulated PDE since the binding of cGMP to the regulatory GAF-B domain of PDE2A enhances the affinity to degrade cAMP (47, 48). With that unique characteristic PDE2A enables the negative cGMP-to-cAMP cross-talk. On the other hand, PDE3 is a cGMP-inhibited PDE because of lower K_m_ value for cGMP. So, the competitive inhibition of cAMP hydrolysis by cGMP binding enables the positive cGMP-to-cAMP cross-talk (49). From a physiological point of view, the cGMP-to-cAMP cross-talk mediated by PDE2 is a key mediator in the heart and the adrenal cortex. For example, the secretion of aldosterone underlies the control of cAMP levels, which is balanced by ANP dependent PDE2A activation in adrenal zona glomerulosa cells (50). The impact of PDE3 in T-cells has been investigated, but so far, less research has focused on PDE2A and the real-time dynamics of cAMP-to-cGMP cross-talk in T-cells. With the use of a highly sensitive Förster Resonance Energy Transfer based sensor it was recently shown that PDE2A inhibition results in higher responses in CD3/CD28-activated than in non-activated T-cells (34). Moreover, PDE2A is selectively upregulated during activation in Tcon but not in Treg. Simultaneously, PDE3B is not upregulated upon CD3/CD28 stimulation. These findings open the question whether the regulation via PDE3B and PDE2A of the cAMP-to-cGMP-cross-talk undergoes a switch by the activation of T-cells (**Figure 1**).

## 6 Conclusion & further perspectives

Intracellular cAMP levels tightly control T-cell function and activation. cAMP is transiently upregulated during T-cell activation, but high levels of cAMP suppress T-cell activation, proliferation, and cytokine release. cAMP levels differ between T-cell subsets and can be transferred via gap junctions to mediate the suppressive function of Tregs. Thus, it is indispensable for the maintenance of the immune balance that cAMP levels in the cell are balanced precisely at a subcellular level by the interplay of cAMP production via ACs and cAMP degradation by PDEs. Research conducted over the last years has focused on PDE4 inhibition to mediate T-cell responses in the context of autoimmune diseases like chronic obstructive pulmonary disorder (COPD), psoriasis and atopic dermatitis. Ongoing research identified the importance of PDE8, alongside the well-characterized PDE4, for T-cell function suggesting it might be a beneficial drug target for multiple sclerosis (46). Moreover, changes in the cGMP-to-cAMP cross-talk could be especially relevant under pathological conditions such as inflammation caused by myocardial infarction where the differentiation of Tregs is promoted (51). High levels of catecholamines and NPs stimulating both cAMP and cGMP signaling at the same time might affect the immune response including the T-cell recruitment to the infarcted tissue. It will be exciting to get a better understanding how dual-substrate PDEs, such as PDE2A and PDE3B, mediate the cGMP-to-cAMP cross-talk to maintain the immune balance under this pathophysiological condition. Furthermore, enhanced PDE2A expression with constant PDE3B expression during activation indicates that the cAMP-to-cGMP cross-talk undergoes a switch during activation. From this perspective, PDE2A could be another important regulator of cAMP in T-cells and potential drug target for immunity and inflammation. Interestingly, PDE2A2 localized in mitochondria can specifically counterbalance local pool of cAMP produced by AC10. Since in several cell types high cAMP levels are known to induce reactive oxygen species production and affect cell apoptosis, similar important role of balanced cAMP signaling can be expected in T cells which can be also affected by autoimmune disease such as multiple sclerosis (52). Therefore, in the future, it will be exciting to study local real-time cAMP dynamics in various subcellular locations in healthy and diseased T-cells.

## Author contributions

MB: Writing – original draft, Writing – review & editing. RK: Writing – review & editing. SF: Writing – review & editing. VN: Writing – original draft, Writing – review & editing.
